# Supplementary material for: Examining the Association between Coffee Intake and the Risk of Developing Irritable Bowel Syndrome: A Systematic Review and Meta-Analysis
Source: Nutrients. 2023 Nov 10;15(22):4745. doi: 10.3390/nu15224745 (PMC10674416; doi:10.3390/nu15224745)
Supplement: Supplementary file 1 [file nutrients-15-04745-s001.zip › nutrients-2691838-supplementary.pdf]

**Supplementary Material**

Table S1. Full search strategy for the various databases

**PubMed**

| | | |
|---|---|---|
| #1 | Irritable bowel syndrome IBS | irritable bowel syndrome[MeSH Terms]<br><br>OR<br><br>("Irritable Bowel"[Text Word] OR "IBS"[Text Word] OR "Irritable Colon"[Text Word] OR "Mucous Colitis"[Text Word] OR "Spastic Colitis"[Text Word] OR "Spastic Colon"[Text Word]) |
| #2 | Coffee | coffee[MeSH Terms]<br><br>OR<br><br>"Coffee"[Text Word] OR "Caffeine"[Text Word] OR "Beverage"[Text Word] |

**Embase**

| | | |
|---|---|---|
| #1 | Irritable bowel syndrome IBS | 'Irritable bowel syndrome'/exp or 'irritable bowel syndrome':ti,ab or 'irritable bowel':ti,ab or 'IBS':ti,ab or 'irritable colon':ti,ab or 'mucous colitis':ti,ab or 'spastic colitis':ti,ab or 'spastic colon':ti,ab |

| | | |
|---|---|---|
| #2 | Coffee | ‘coffee’/exp or ‘coffee’:ti,ab or ‘caffeine’:ti,ab or ‘beverage’:ti,ab |

**Cochrane Library**

| | | |
|---|---|---|
| #1 | Irritable bowel syndrome<br>IBS | MeSH descriptor: [Irritable Bowel Syndrome] explode all trees<br><br>OR<br><br>(“irritable bowel syndrome” or “irritable bowel” or “IBS” or “irritable colon” or “mucous colitis” or “spastic colitis” or “spastic colon”):ti,ab,kw |
| #2 | Coffee | MeSH descriptor: [Coffee] explode all trees<br><br>OR<br><br>(“coffee” or “caffeine” or “beverage”):ti,ab,kw |

Table S2. Newcastle Ottawa Scale for cross-sectional studies

| Study | Author | Year | Selection Bias Assessment (Maximum 4 stars) | | | | Comparability (Maximum 2 stars) | | Outcome (maximum 3 stars) | | |
|---|---|---|---|---|---|---|---|---|---|---|---|
| | | | Representativeness of the sample | Selection of control group | Ascertainment of exposure | Non-respondents | Main factor | Additional factor | Assessment of outcomes | Statistical test | Total (10/10) |
| 1 | Khademolhosseini et al[7] | 2011 | * | * | * | 0 | 0 | 0 | * | * | 5/10 |
| 2 | Basandra et al[8] | 2014 | 0 | * | 0 | 0 | 0 | 0 | * | * | 3/10 |
| 3 | Al Saadi et al[24] | 2016 | 0 | * | * | 0 | 0 | 0 | * | * | 4/10 |
| 4 | Kesuma et al[25] | 2021 | * | * | * | * | 0 | 0 | * | * | 6/10 |
| 5 | Koochakpoor et al[26] | 2021 | * | * | * | 0 | * | * | * | * | 7/10 |
| 6 | El Sharawy et al[27] | 2022 | 0 | * | * | 0 | 0 | 0 | * | * | 4/10 |

## Table S3. Newcastle Ottawa Scale for case-control studies

| Study | Author | Year | Selection Bias Assessment (Maximum 4 stars) | | | | Comparability (Maximum 2 stars) | | Exposure (maximum 3 stars) | | | |
|---|---|---|---|---|---|---|---|---|---|---|---|---|
| | | | Is the case definition adequate? | Representativeness of the cases | Selection of controls | Definition of controls | Main factor | Additional factor | Ascertainment of exposure | Same method of ascertainment for cases and controls | Non-response rate | Total (9/9) |
| 1 | Guo et al[8] | 2015 | * | * | * | * | * | * | * | * | * | 9/9 |

## Table S4. Newcastle Ottawa Scale for cohort studies

| Study | Author | Year | Selection Bias Assessment (Maximum 4 stars) | | | | Comparability (Maximum of 2 stars) | | Outcome (maximum 3 stars) | | | |
|---|---|---|---|---|---|---|---|---|---|---|---|---|
| | | | Representativeness of the exposed cohort | Selection of the non-exposed control | Ascertainment of exposure | Outcome of interest not present at the start of the study | Age, sex, marital status | Additional factor | Assessment of outcomes | Sufficient follow-up time | Adequacy of follow-up | Total (9/9) |
| 1 | Wu et al[28] | 2023 | * | * | * | * | 0 | * | * | * | 0 | 7/9 |
